# The Impact of Optical Berry Sorting on Red Wine Composition and Sensory Properties

**DOI:** 10.3390/foods10020402

**Published:** 2021-02-12

**Authors:** Robert C. Bruce, Pauline Lestringant, Charles A. Brenneman, Hildegarde Heymann, Anita Oberholster

**Affiliations:** Department of Viticulture and Enology, University of California, Davis, CA 95616, USA; rcbruce@ucdavis.edu (R.C.B.); plestringant@ucdavis.edu (P.L.); cabrenneman@ucdavis.edu (C.A.B.); hheymann@ucdavis.edu (H.H.)

**Keywords:** grapes, wine, optical berry sorting, phenolics, volatiles, descriptive analysis

## Abstract

The impact of optical berry sorting was investigated using Grenache, Barbera, and Cabernet Sauvignon grapes from Yolo County, California in 2016. Optical sorting parameters were adjusted to remove underripe berries and material other than grapes using color parameters. Wines were made from three treatments, control (no sorting), sort (accepted material), and reject (material rejected by the optical sorter). The rate of rejection was approximately 14.9%, 3.9%, and 1.5% (*w*/*w*) for Grenache, Barbera, and Cabernet Sauvignon, respectively. Chemical composition in the finished wines was analyzed by the Adams-Harbertson assay and reversed-phase high-performance liquid chromatography for phenolics, and head-space solid-phase microextraction gas chromatography mass spectrometry for aroma profiling. In general, optical sorting was successful in removing underripe berries and material other than grapes as evidenced by lower ethanol levels and higher concentrations of total phenolics and tannin (due to the inclusion of material other than grapes) in wine made from rejected material. Despite this, no difference in final ethanol content and minimal differences in phenolic composition were observed between control and sort treatment wines for the three varieties studied. Differences were observed in the aroma profiles of the reject treatments for all three varieties compared to sort and control; however, few compounds differed significantly between the sort and control treatments. Descriptive sensory analysis revealed that panelists had difficulty distinguishing aroma, taste, mouthfeel, and color parameters among wines made from different treatments for all three varieties. Thus, optical sorting had minimal impact on wine sensory properties using the varieties and vineyards studied. Optical sorting may be used to differentiate and sort for different ripeness levels using color as a primary criterion; however, the impact on the resulting wine is likely dependent on the initial variability in grape ripeness.

## 1. Introduction

In an effort to improve wine quality, many smaller high-end wineries employ laborers to hand sort individual berries after destemming to remove unwanted material such as raisins, diseased berries, unripe berries, and materials other than grapes (MOG) such as leaves and stems. This can be costly, labor intensive, and it can slow down the process line. To reduce costs and increase throughput, many wineries have adopted optical sorting technology. Using this technology, MOG can be removed more efficiently, and parameters such as color, shape, and size can be used to sort individual berries. Depending on the type of sorter, processing speeds can range between 2 and 15 tons per hour. Furthermore, fewer workers are needed to operate an optical sorter than to hand sort the respective amount of fruit [1]. 

In addition to saving time and money, optical sorters have the potential to decrease the impact of inconsistent ripening in grapes. One study successfully sorted Carlos Muscadine grapes into four different ripeness levels using light at two different wavelengths in the visible spectrum [2]. The researchers found that with successive sorting levels, there was an increase in Brix and pH, along with a decrease in titratable acidity in grape samples. In the wines, an increase in tannin and pH and a decrease in titratable acidity was found with increasing sorting. In sensory analysis, the first and fourth sorting levels were found to be inferior compared to the middle two treatments. Even though this study used outdated equipment compared to today’s standards, it shows that white grapes can be sorted into different ripeness levels and this can affect the quality of the wine produced. A recent study used visible near-infrared spectroscopy to classify table grapes into different groups based on soluble solid and phenolic content [3]. The researchers were able to differentiate berries of different classes with accuracy ranging from 77% to 94%. Another study found that wine made from optically sorted Chardonnay grapes had higher residual sugar, pH, and total phenols than the unsorted control [4]. The wines were analyzed sensorially with descriptive analysis and the judges scored the sorted wines significantly higher in tropical fruit and sweetness. However, with only two significant attributes out of twenty, the wines were determined to be similar in character. Another study investigating the effect of mechanical harvesting and optical berry sorting on Pinot noir grapes found that, in general, wines made from optically sorted fruit were significantly lower in total phenol and tannin, potentially due to the removal of MOG during sorting [5]. In sensory analysis only two significant attributes out of eighteen were found (tropical fruit and hue saturation) and it was concluded that the wines were similar in character. A study published in 2014 used an optical sorter on Riesling, Müller-Thurgau, and Pinot gris grapes infected with Botrytis cinerea to investigate the effect of optical sorting on sulfur binding compounds in the finished wine [6]. The researchers found that wine made from optically sorted fruit contained significantly less 2-oxoglutaric acid and pyruvic acid (both are common sulfur binding compounds that can be higher in wines made from grapes infected with Botrytis cinerea). They concluded that optical sorting is an effective method for reducing the amount of sulfur dioxide (SO_2_) needed in the winemaking process using these varieties. There is a lack of published research investigating the impact of optical berry sorting on wine composition and only a few cultivars of *Vitis vinifera* have been tested. The objective of the current study was to provide more information on the effect of optical berry sorting on different varieties and investigate the capabilities of today’s optical sorters to sort for different ripeness levels using red grapes and using color as a sorting parameter. The current study found that although optical sorting can efficiently replace hand sorting, the overall impact on wine sensory attributes was minimal. Therefore, in general, the study supported the findings of previous researchers. 

## 2. Materials and Methods 

Three varieties were tested in 2016: Barbera (BA), Cabernet Sauvignon (CS), and Grenache (GN). BA was harvested on 19 August 2016, CS was harvested 30 August 30 2016, and GN was harvested 8 September 2016. All three varieties were hand harvested early in the morning from UC Davis campus vineyards and processed the same day. Fruit condition was good with seemingly little variation, although GN fruit showed more variation in color than the other cultivars. Half-ton bins were dumped by a forklift into a receiving hoper. Clusters were carried by a Delta TR elevator (Bucher-Vaslin, Santa Rosa, CA, USA) into a Delta E2 destemmer (Bucher-Vaslin, Santa Rosa, CA, USA). Destemmed berries fell onto a moving belt and were carried onto a ChromaxHD Berrytek Optical Sorter (Woodside Electronics Corporation, WECO, Woodland, CA, USA). Rejection parameters were established by capturing color profiles of optimal berries, suboptimal berries (green/underripe berries and raisins), and MOG. These parameters were optimized with the assistance of a WECO technician for removing suboptimal berries and MOG while rejecting as few optimal berries as possible. This process was repeated, and parameters were adjusted for each variety. The must was pumped directly into 200 L stainless steel research fermentors [7], which were filled incrementally to reduce vineyard variation. The rejected material was collected in buckets and transferred into research fermentors. The grapes were processed in three treatments, control (no sorting), sort (accepted material), and reject (material rejected by the optical sorter). The rejection rates were 14.9%, 3.9%, and 1.5% (*w*/*w*) for GN, BA, and CS, respectively. Juice collected in trays from the rolling belts during processing operations was added back to each treatment in proportional amounts. This was done to maintain a consistent solid-to-juice ratio in the must among treatments.

Wines were made in the UC Davis Teaching and Research Winery using 200 L stainless steel research fermentors. The control and sort treatments were fermented in triplicate and the reject treatments were fermented in duplicate. Duplicate fermentations were used for the reject treatment wines because only a small amount of reject material was obtained during grape processing. Fermentation replications were kept separate through the entire experiment. Juice samples were taken from each fermentation vessel after mixing. Fifty milligrams per liter of SO_2_ was added to the must after processing using a 15% potassium metabisulfite solution. Yeast assimilable nitrogen (YAN) was adjusted to 250 mg/L using diammonium phosphate (DAP), titratable acidity (TA) was adjusted (if necessary) to 6 g/L using tartaric acid. The must was heated to 25 °C before inoculation with Lalvin EC1118 yeast (Lallemand, Inc., Petaluma, CA, USA) using the manufacturers rehydration procedure. One tank volume was pumped over twice per day using automated pump overs for all wines except for the reject treatment for CS. The volume in these tanks was too low for the automated pumps to create suction, therefore, the wines were punched down manually once per day during the fermentation. Once wines were dry, they were pressed using a basket press and allowed to settle for 5 days before being racked and transferred to a temperature-controlled room held at 20 °C. The wines were then inoculated with Viniflora CH16 *Oenococcus oeni* bacteria by Chr. Hansen (Milwaukee, WI, USA). Upon finishing the malolactic (ML) fermentation, 50 mg/L SO_2_ was added to the wines and they were held in a 9 °C cold room until bottling. Free SO_2_ was adjusted to 30 mg/L before bottling. All samples for basic wine chemical analyses were taken at the time of bottling. Ethanol (*v*/*v*) was measured using an Alcolyzer (Anton Paar, Ashland, VA, USA) and pH was measured using an Orion 5-star pH meter (Thermo Scientific, Waltham, MA, USA). Titratable acidity (TA) and free SO_2_ were measured using a Mettler Toledo DL50 auto titrator (Mettler Toledo, Columbus, OH, USA). Residual sugar, malic acid, and volatile acidity were measured using a Thermo Fisher Scientific Gallery automated analyzer (Thermo Scientific, Waltham, MA, USA). Wines were sterile filtered using 0.45 μm membrane filters (Millipore, Burlington, MA, USA) prior to bottling using green Bordeaux style bottles and screw cap closures with Saranex liners by Amcor (Yuba City, CA, USA). 

Samples for phenolic analysis were taken from bottles at the time of sensory analysis. The modified Adams-Harbertson [8] assay was used to determine levels of anthocyanin (expressed as malvidin-3-glucoside equivalents), tannin (expressed as catechin equivalents), and total iron-reactive phenolics (expressed as catechin equivalents) [8]. A Genesis 10S UV-Vis spectrophotometer (Thermo Scientific, Waltham, MA, USA) was used for this assay.

Phenolic compounds were also analyzed by a method using reverse-phase high-performance liquid chromatography (RP-HPLC) previously described in the literature [9]. Briefly, compounds were measured at four wavelengths; 280 nm (gallic acid, (+)-catechin, dimer B1, (−)-epicatechin, epicatechin gallate, and polymeric phenols); 320 nm (caftaric acid, caffeic acid, coutaric acid, and p-coumaric acid); 360 nm (quercetin-3-galactoside, quercetin-3-glucoside, quercetin-3-glucuronide, quercetin-3-rhamnoside, and quercetin); and 520 nm (delphinidin-3-glucoside, petunidin-3-glucoside, peonidin-3-glucoside, malvidin-3-glucoside, malvidin-3-acetylglucoside, malvidin-3-p-coumglucoside, and polymeric pigments). Wine samples were stored in HPLC vials in a −20 °C freezer until analysis. An Agilent 1260 Infinity HPLC (Agilent Technologies, Santa Clara, CA, USA) with a diode array detector (DAD) was used. An Agilent PLRP-S (150 × 4.6 mm, 100 A, and 3 µm pore size) column with an Agilent PLRP-S guard cartridge (5 × 3 mm) was maintained at 35 °C. Agilent CDS ChemStation software (version B.04) was used for instrument control and data analysis. The injection volume was 20 µL and a gradient mobile phase of water with 0.3% phosphoric acid (*v*/*v*) (88%, Fisher Scientific, Pittsburgh, PA, USA) (mobile phase A) and acetonitrile (HPLC grade, Sigma-Aldrich, St. Louis, MO, USA) with 0.2% phosphoric acid (*v*/*v*) (mobile phase B) was used at a flow rate of 1.0 mL/min. The solvent gradient was 6–31% B at 0–73 min, 31–62% B at 73–78 min, isocratic 62% B at 78–86 min, 62–6% B at 86–90 min. Compounds were identified using retention time and spectral comparison to standards. An external calibration was used for the quantification of phenolic compounds and curves were made for gallic acid, (+)-catechin (98%, Sigma-Aldrich, St. Louis, MO, USA), (−)-epicatechin (95%, Sigma-Aldrich, St. Louis, MO, USA), caffeic acid (98%, Sigma-Aldrich, St. Louis, MO, USA), quercetin-3-rhamnoside, and malvidin-3-glucoside. Caftaric acid was quantified as caffeic acid equivalents, quercetin-glycosides as quercetin-3-O-rhamnoside (98.5%, Indofine Chemical Company, Hillsborough, NJ, USA) units and all pigments as malvidin-3-glucoside (95%, Extrasynthese, Genay, France) units. Bottle duplicates for each fermentation replication were analyzed and the sequence was randomized.

Wine aroma compounds were analyzed using head-space solid-phase microextraction gas chromatography mass spectrometry (HS-SPME-GC-MS). The method used was adapted from a previous study [5]. Samples used for wine volatile analysis were taken at the time of sensory analysis and stored at 4 °C for no more than one month. Identified volatile peaks are normalized against an internal standard and the obtained data is thus semiquantitative only. Twenty mL amber glass headspace vials (Agilent Technologies) were used, containing 10 mL milliliters of wine sample, 3 g of NaCl salt and 50 µL of a 10 mg/L solution of 2-undecanone (internal standard, 99%, Sigma-Aldrich, St. Louis, MO, USA). Twenty millimeter green magnetic caps with 3 mm PTFE silicone septa (Supelco, St. Louis, MO, USA) were crimped onto the vials and the samples were mixed by vortexing. The analysis was done using an Agilent Technologies 7890A GC system (Agilent Technologies, Santa Clara, CA, USA) with a Gerstel MPS2 multipurpose sampler (Mülheim an der Ruhr, Deutschland). The mass analyzer was an Agilent Technologies 5975C inert XL EI/CI MSD. The column used was an Agilent Technologies DB-Waxetr with a temperature range of 30 °C to 260 °C. The dimensions of the column were 30 m, 0.250 mm, and 0.25 µm. Maestro software (version 1.2.3.1; Gerstel) was used to control the instrument and data were collected using ChemStation software (version E.01.01.335; Agilent). During the analysis, the oven was held at 40 °C for 5 min and then increased 3 °C/min to 180 °C, followed by 30 °C/min to 250 °C and held for 7.67 min. The MSD interface was kept at 260 °C. HS-SPME-GC-MS conditions were as previously described [5]. Shortly, samples were heated to 30 °C for five minutes while agitating with a speed of 500 rpm prior to exposing the fiber (1 cm polydimethylsiloxane 23-gauge SPME fiber, Supelco, St. Louis, MO, USA) to the sample for 45 min at 30 °C with agitation at 250 rpm. The SPME fiber was desorbed in split mode with a 10:1 split ratio and the inlet temperature was kept at 260 °C. Bottle duplicates were analyzed in triplicate for each treatment. Compound details are provided in Table S1.

Wines were analyzed sensorially using descriptive analysis in the J. Lohr Wine Sensory Room, University of California, Davis, CA. GN, BA, and CS wines were analyzed approximately two, three, and four months, respectively, after bottling. Three separate descriptive analysis panels were utilized, one for each variety. Eleven panelists were recruited for GN, and ten each for BA and CS. The panelists were offered $30 gift certificates for completion of the study. The study was approved by the International Review Board (571923-1) and all participants reviewed and agreed to the terms of the experiment. None of the panelists knew details of the experiment.

Two fermentation replicates were selected from each treatment totaling six wines for each descriptive analysis study. There were six training sessions and three evaluation sessions. The panelists were given 30 mL of each wine sample for both the training sessions and the evaluation sessions. The wines were presented blind using black wine glasses (ISO 3591:1977) and the order was randomized for each session. In the first training session, panelists generated descriptors used for differentiating the wines. In subsequent sessions, the reference standards for each descriptor were optimized through panel discussions until there was general agreement. The list of descriptors was narrowed down until there were twenty descriptors for GN (12 aroma, 4 taste, and 4 mouthfeel), twenty-six for BA (14 aroma, 4 taste, and 8 mouthfeel), and twenty-two for CS (11 aroma, 5 taste, and 4 mouthfeel; Table 1, Table 2 and Table 3). Panelists were asked to rate the intensity of each attribute using an unmarked line scale. Reference standards were given as an anchor for the high end of the intensity scale of each attribute. In addition to these attributes, panelists also analyzed color by matching each wine with a color chart (Les Couleurs Du Vin, Bouchard Aîné & Fils). Panelists were given 30 mL of wine in a clear glass and instructed to hold the glass at arm’s length with a white background and match with the closest color on the poster. Scores were reported by assigning number values to each color on the poster. Perceived color differences from sensory analysis were compared to wine colors determined using a CR-400 Chroma Meter (Konica Minolta, Ramsey, NJ, USA) using the CIELAB color space.

Wines were analyzed by the panelists in triplicate using a randomized block design over a one-week period. All analyses were completed in isolated booths with positive air flow and red lighting. Randomized three-digit codes were assigned to the wines (unique for each panelist for each session) to eliminate biases. Panelists were given breaks in between each wine and were encouraged to drink water and eat an unsalted cracker as a pallet cleanser. All samples were expectorated. Data were collected using FIZZ software (version 2.00L, Biosystems, Dijon, France). 

All statistical analyses were carried out using XLSTAT (Microsoft Office Professional Plus 2010, version 14.0.7194.5000, Redmond, WA, USA). Univariate analysis of variance (ANOVA) was used for all data in determining significant differences. For descriptive analysis data, multivariate analysis of variance (MANOVA) was used prior to ANOVA to determine the main treatment effect. ANOVA was used for judge, treatment, and replicate effects along with a pseudo mixed model. Fisher’s least significant difference (LSD) was used for pairwise comparisons of means. Statistical significance was set at 5% for all tests. 

## 3. Results

### 3.1. Juice and Wine Chemistry

Analysis of Brix, pH, and TA of the musts showed minimal differences among treatments for each variety (Table 4). There were no significant differences for all three parameters of the BA must and only the reject treatment for GN had a significantly higher TA; however, this difference was not large. It is possible that this difference could be the result of the inclusion of underripe berries in the must, which have a higher TA. Raisins were also rejected from the sorter, which are high in sugar and could have compensated for the difference in sugar from the less ripe berries. The CS must exhibited the most differences among treatments, which was unexpected considering this variety had the lowest percentage of rejected fruit (1.5%, *w*/*w*, compared to 14.9% and 3.9% for GN and BA respectively). The Brix was significantly higher in the sorted treatment compared to the control and reject treatments. This may indicate that the sorter was effective at removing less ripe berries for CS. The pH also differed significantly among treatments for CS; pH was highest in the reject must at 3.8, followed by sort and control at 3.71 and 3.67 respectively. Although the difference in pH between the sort and control was statistically significant, they are very similar with only a 0.04 pH unit difference. Overall, the differences seen in the must chemistry were minimal and likely made little to no difference in the progression of the wines. It is possible that the reject must composition was made to be more similar to the control and sort treatments due to the addition of juice that accumulated in the vibrating table trays. If this was not done perhaps there would be more differences in must composition when comparing the reject to the sort and control treatments.

Wine chemical compositions are shown in Table 5. All wines progressed consistently through fermentation and fermented dry with less than 1 g/L residual sugar. For the most part, wine chemical compositions are similar among treatments for each variety, especially between the sort and control treatments. However, there are some important exceptions. Ethanol content for the BA and CS wines was significantly lower in the reject treatments. This mostly corresponds to differences in the starting sugar content, although there is a discrepancy as BA reject wines were not significantly lower in Brix. However, Brix was determined after mixing of the must, and especially if a significant number of raisins were present, soak up in the next 24 h could have resulted in sugar increases. The malolactic fermentations for GN and CS wines progressed to completion; however, the control and sort treatments for BA did not finish and were left with close to 1 g/L malic acid for each of the treatments (data not shown). This is likely due to the high ethanol content in addition to high TA in the wines which can inhibit malolactic bacteria [10]. This would also explain why the reject treatment for BA progressed further in the malolactic fermentation given that these wines were lower in ethanol content and TA. This difference could have important implications for the sensory analysis of BA wines. The volatile acidity (VA) for the CS reject treatment was significantly higher than that for the control and sort treatments (0.6 g/L compared to 0.2 g/L for the sort and control). The sensory threshold has been reported to be approximately 0.8 g/L for red table wines, therefore, this discrepancy may not have a large impact on sensory analysis [11]. It was surprising that GN musts/wines showed few significant differences despite having the largest rate of rejection (14.9%). It is possible that sorting parameters were too aggressive when processing GN, which may have inadvertently led to the rejection of optimal fruit. As previously mentioned, there was significant variation in color for GN fruit (especially if the cuticle was removed from the berry skin). It may also be possible that observed color difference in GN fruit did not correlate well with sugar content. This would mean that optical sorting based on color for GN fruit from this vineyard is potentially less effective than for the other varieties.

### 3.2. Wine Phenolics

Differences among treatments were observed in total phenolics, tannin, and anthocyanin content as measured by the Adams-Harbertson assay (Table 6). In general, the reject wines were higher than control and sort wines in total phenolics and tannin, and lower in anthocyanin. This may be explained by the inclusion of MOG in the reject fermentations which can lead to greater extraction of phenolics [12]. Lower anthocyanin levels were observed in the reject wines for all varieties. This is most likely due to the inclusion of green, underripe berries, which contain less anthocyanin. 

In general, the results from the RP-HPLC analysis of phenolics agree with the results obtained from the Adams-Harbertson assay (Table 7, Table 8 and Table 9). Higher levels of most phenolic compounds were observed in the reject treatments. Concentrations of gallic acid and catechin were higher in the reject treatments for all three varieties and dimer B1 was higher in reject wines for BA and CS. Less ripe berries have been shown to contain more of these compounds, which can explain this trend [13]. Higher levels of identified flavan-3-ols were also observed in the reject treatments of BA and CS wines, which is also in agreement with results found by Obreque-Slier [13]. An interesting trend was found in relation to the proportions of simple hydroxycinnamic acids and their respective tartaric acid esters. All the reject treatments had very low amounts of caftaric and coutaric acid compared to caffeic and p-coumaric acid. It is possible that hydroxycinnamoyl esterase, the enzyme responsible for hydrolyzing the ester linkage, had a greater activity in the reject wines, possibly due to differences in pH [14]. Another possibility is that there could be higher levels of this enzyme in less-ripe fruit. The reject wines for all three varieties were also significantly lower in anthocyanin, which matches results obtained by the Adam-Harbertson assay. Although reject wines had higher levels of most phenolic compounds, this did not lead to large differences between sorted and control wines. It is likely that not enough material (MOG and green berries) was removed during processing for there to be a significant effect. This may also explain why there were no significant differences in anthocyanin content between sorted and control wines despite reject wines being significantly lower. Perhaps a greater effect would be observed with more aggressive sorting parameters and/or fruit with more variability. Overall, the levels of most phenolic compounds identified were very similar between the sort and control treatments. It can be concluded that optical sorting had little impact on the composition of phenolic compounds between sorted and control wines in all three varieties tested.

### 3.3. HS-SPME-GC-MS Analysis of Wine Volatiles 

**Figure 1:**
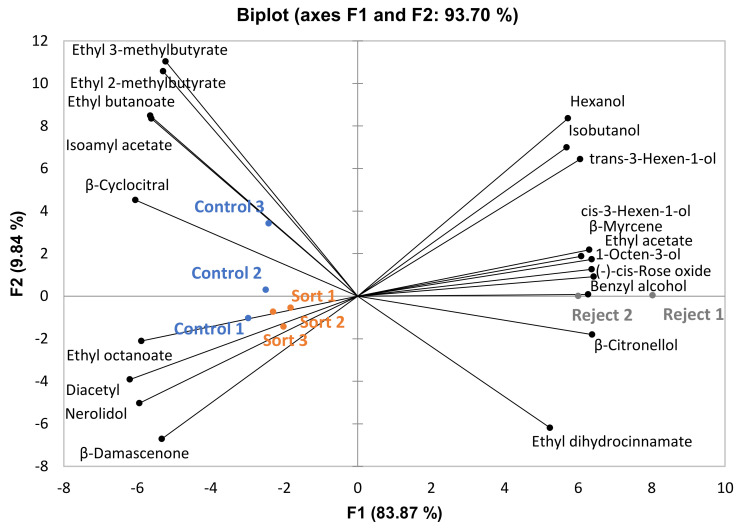
Principle Component Analysis (PCA) biplot of volatile compounds that differed significantly among treatments for the Cabernet Sauvignon wines (*p* < 0.05, *n* = 3 for sort and control treatments, *n* = 2 for reject treatment).

For CS wines, 37 volatile compounds were identified, 20 of which differed significantly among treatments (*p* < 0.05, *n* = 3 for sort and control, *n* = 2 for reject); however, only one compound differed significantly between wines made from sorted and control treatments (β-citronellol was significantly higher in control wines). A Principle Component Analysis (PCA) biplot plot of significant compounds is presented in Figure 1. It appears the separation is driven primarily by ethyl esters (ethyl 3-methylbutyrate, ethyl 2-methylbutyrate, ethyl butanoate, and ethyl octanoate) on the left and higher alcohols (hexanol, isobutanol, trans-3-hexen-1-ol, cis-3-hexen-1-ol, and 1-octen-3-ol) on the right. Most ethyl esters have higher concentrations in wines made from control and sort treatments (Figure 1). Esters in wine can be formed by an acid catalyzed esterification reaction between an acid and alcohol [15]. 

Higher amounts of either acid or alcohol can result in increased formation of esters. Wines made from control and sorted treatments had higher ethanol content (Table 5) than wines made from the reject treatment, which would explain this trend. Another important trend is the association of reject treatment wines with a larger concentration of higher alcohols. The suspended solids concentration was significantly higher in reject treatment musts (measured in Nephelometric Turbidity Units, data not shown) which may explain the difference in the concentration of higher alcohols among the treatments, as previous research has shown that suspended solids during fermentation can lead to greater production of higher alcohols [16,17,18].

**Figure 2:**
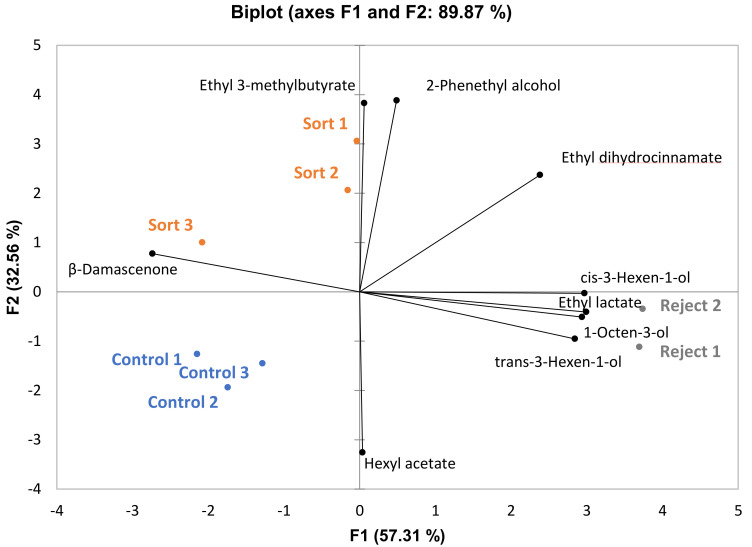
PCA biplot of volatile compounds that differed significantly among treatments for the Barbera wines (*p* < 0.05, *n* = 3 for sort and control treatments, *n* = 2 for reject treatment).

PCA loading and score plots of volatile analysis for BA wines are given in Figure 2. Thirty-seven compounds were identified, and nine differed significantly among treatments (four differed significantly between wines made from sort and control treatments). Again, separation seems to be driven by the proportionally larger presence of higher alcohols in the reject treatments. Like the CS reject musts, the BA reject musts also had significantly higher levels of soluble solids compared to the sort and control treatments (data not shown). Although most ethyl esters did not differ significantly among treatments for BA, there was a general trend indicating that ethyl ester content was higher in the control and sort treatments (data not shown). A PCA biplot using all identified ethyl esters and higher alcohols is provided in Figure S1 and there is a clear trend in the separation of these compounds. This agrees with the previous discussion regarding ethyl ester content in the CS wines. The BA control and sort treatments had significantly higher ethanol content (Table 5) compared to the rejects so it is expected that ethyl ester concentration would be higher as well. One exception to this trend is that ethyl lactate was significantly higher in the reject treatment. The reject wines completed ML fermentation, but the control and sort wines got stuck with almost 1 g/L malic acid (data not shown). Therefore, ethyl lactate is significantly higher in reject wines because there was more lactic acid present from the conversion of nearly all the malic acid. 

**Figure 3:**
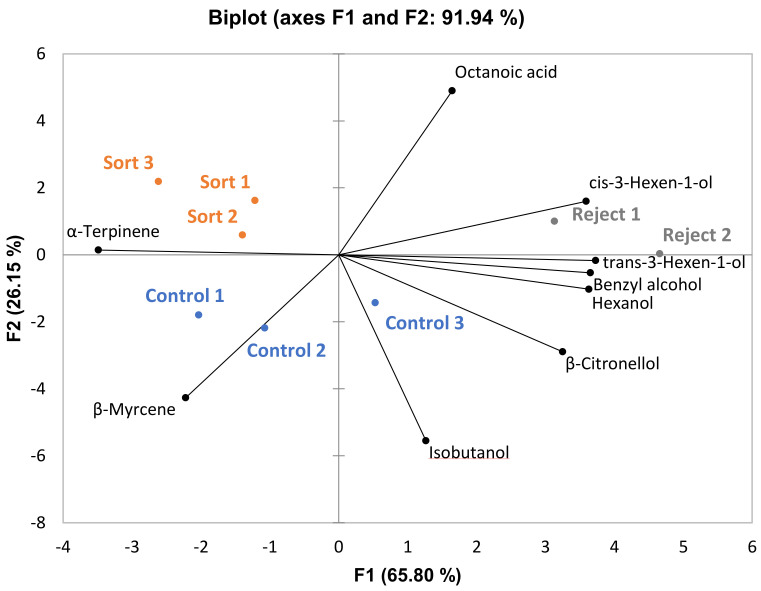
PCA biplot of volatile compounds that differed significantly among treatments for the Grenache wines (*p* < 0.05, *n* = 3 for sort and control treatments, *n* = 2 for reject treatment).

For GN wines, 32 compounds were identified, nine of which differed significantly among treatments and four differed significantly between sort and control treatments. The same trend was observed for higher alcohols for GN as for the other varieties driving separation in the PCA plot (Figure 3). The concentrations of cis-3-hexen-1-ol, trans-3-hexen-1-ol, and hexanol were all significantly higher in the reject treatments. Again, this is most likely due to higher suspended solids content in the reject treatment musts (data not shown). The trend with ethyl esters was not observed for GN wines, most likely because all treatments had similar ethanol content (Table 5). Overall, the results indicate that optical sorting had a minimal effect on the aroma profile for all three varieties, particularly when comparing sort and control treatments.

### 3.4. Descriptive Analysis

**Figure 4:**
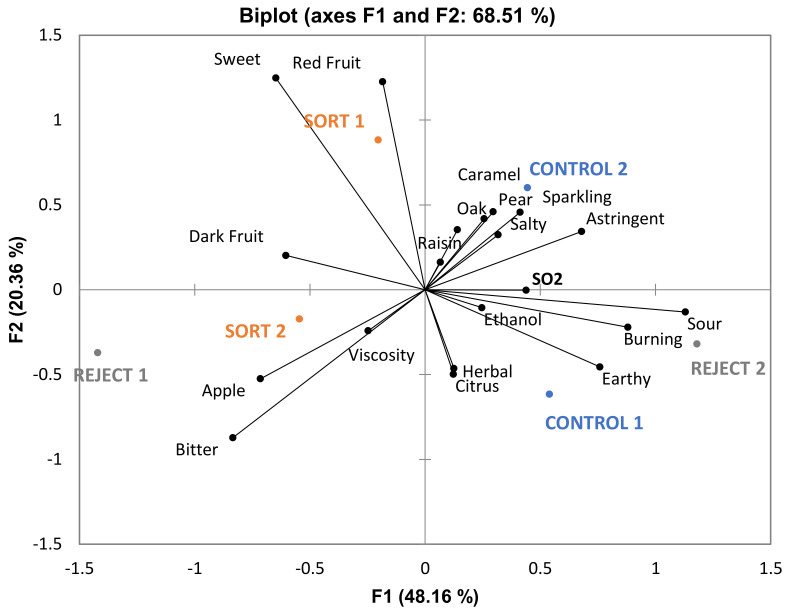
Biplot PCA of Grenache wines generated by descriptive sensory analysis. Significant attributes are given in bold (*n* = 11, *p* < 0.05).

Given the uniformity of chemical results among biological replications, it is fair to assume that the two replications used for descriptive analysis are representative, and the chemical results can therefore be used to discuss sensory trends. MANOVA was performed and revealed a nonsignificant treatment effect for all three varieties (Tables S2–S4, *p* < 0.05). From this result, it can be concluded that all three treatments for each variety were similar in sensory properties. Despite this result, ANOVA was carried out on individual attributes and some significant differences were found for each variety (cobweb plots are provided in Figures S2–S4). For GN, only one attribute (“SO2”) out of twenty differed significantly among treatments. It is possible that sensory analysis was done too soon after the wines were bottled (GN wines were bottled two months before sensory analysis) and the levels of molecular sulfur dioxide may have been above sensory threshold of about 2 mg/L [15]. Figure 4 gives a PCA biplot with all attributes from the GN descriptive analysis panel. There are no clear trends from the PCA; therefore, it can be concluded from MANOVA, ANOVA, and PCA results that all treatments lead to wines of similar character for GN wines.

**Figure 5:**
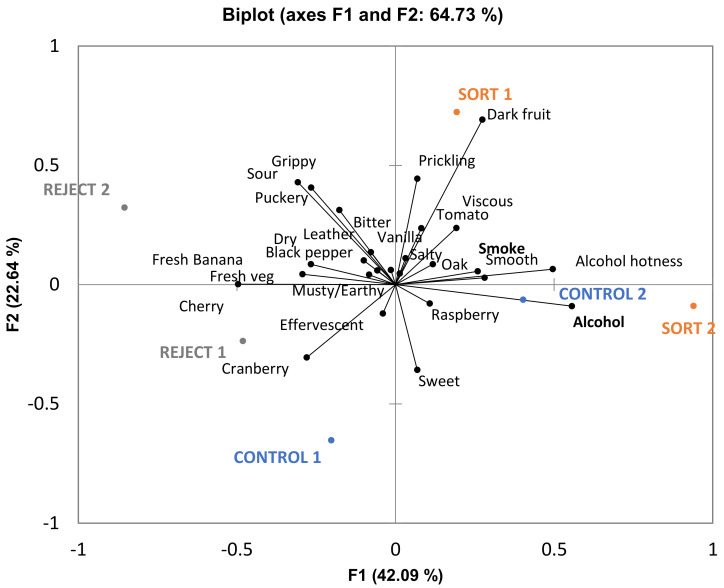
Biplot PCA of Barbera wines generated by descriptive sensory analysis. Significant attributes are given in bold (*n* = 10, *p* < 0.05).

When ANOVA was performed on data from the BA descriptive analysis panel, three out of twenty-six attributes (“smoke”, “alcohol” and “alcohol hotness”) were found to be significantly different among treatments. “Alcohol hotness” (describing alcohol hotness in the mouth) had a significant judge-by-treatment interaction. Results from the pseudo mixed model indicated the interaction effect was more important than the treatment effect. Thus, “alcohol hotness” will not be included in any further discussion of significant attributes for BA wines. The significant difference in malic acid content in the wines among treatments appears to have had little impact on sensory evaluation given that there was no significant difference in the perception of sourness in the wines. From the PCA generated from BA descriptive analysis results (Figure 5), the control and sort wines appear to be correlated more closely with “alcohol” (describing alcohol aroma). Wines made from these treatments were higher in ethanol content, which may explain this trend. However, the small number of significant attributes indicate that BA wines made by different treatments were very similar in sensory properties.

**Figure 6:**
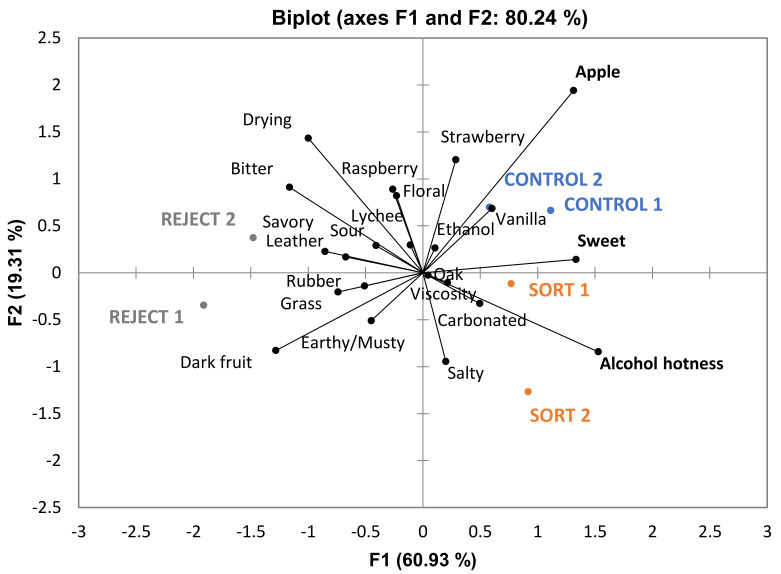
Biplot PCA of Cabernet Sauvignon wines generated by descriptive sensory analysis. Wine treatments as well as significant attributes are given in bold (*n* = 10, *p* < 0.05).

For the CS descriptive analysis panel, three out of twenty-two attributes (“apple”, “sweet”, and “alcohol hotness”) were found to be significantly different when ANOVA was performed. A PCA biplot from the CS panel is provided in Figure 6. The wines made from control and sort treatments are more closely associated with each of the significant attributes; this trend generally matches the results from the chemical analyses. Both the wines made from control and sort treatments were higher in ethanol content, which can explain their greater association with “alcohol hotness” (mouthfeel) when compared to reject treatment wines. It is possible that the higher ethyl ester concentration in the control and sort treatment wines could explain why they are rated significantly higher in the “apple” aroma. Most ethyl esters have fruity aromas which the judges could have rated as “apple”. Curiously, the control and sort treatment wines are rated significantly higher in “sweet” as well despite the residual sugar content of all wines being less than 1 g/L (Table 5), which is below the sensory threshold (reported values of around 1.8–4.0 g/L; [19]). All three significant attributes for the CS panel were rated similarly between control and sort treatment wines. This suggests that these wines made from these treatments had similar sensory properties.

Analysis of wine color revealed that there were perceivable differences among treatments for all three varieties (Table 10). For BA the reject treatments were rated lighter in color compared to the control and sort treatments, whereas a similar trend was observed in the CS treatments. This was expected because berries with less color were removed by the optical sorter and included in the reject fermentations. This agrees with results from Table 6; the rejected treatments were significantly lower in anthocyanin content for BA and CS, which can explain the difference in color perception. For GN wines, the control treatment was perceived to be slightly darker than the sort and reject treatments. Although fermentations were prepared to have similar solid-to-juice ratios in the must among treatments, it is possible that variations between replicates may have resulted in the control treatments being slightly more concentrated, which could provide an explanation for this result. Color perception from the panelists matches well with the wine color determined in the CIELAB color space (Table 11). It can be concluded that optical sorting was generally successful in removing berries with less color; however, this did not lead to a large difference in the final color of the wines between the sort and control treatments.

**Figure 7:**
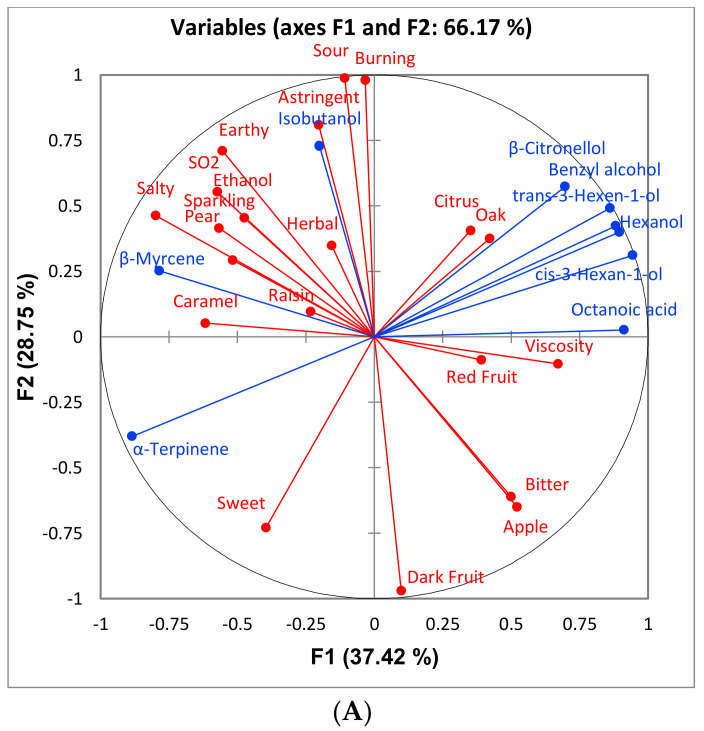

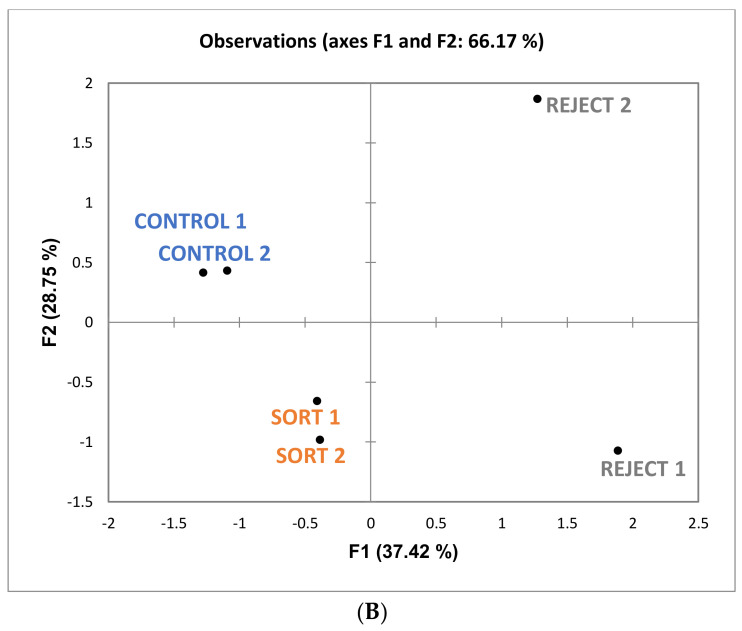
Loadings (**A**) and score (**B**) plots of a multiple factor analysis (MFA) between volatile compounds (blue) in Grenache wines that differed significantly, and all sensory attributes (red) generated from the respective descriptive analysis panel. Only biological replications consistent with those for sensory were included in the MFA.

**Figure 8:**
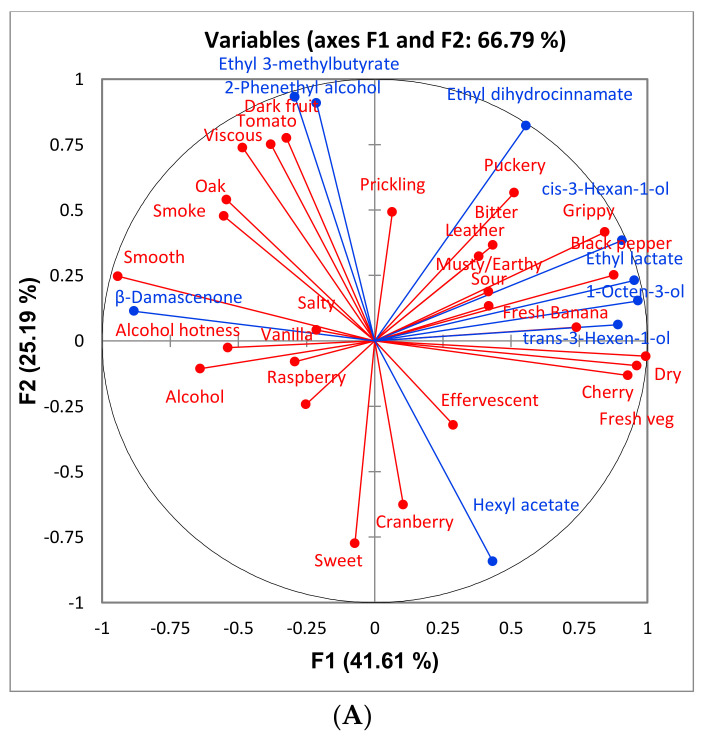

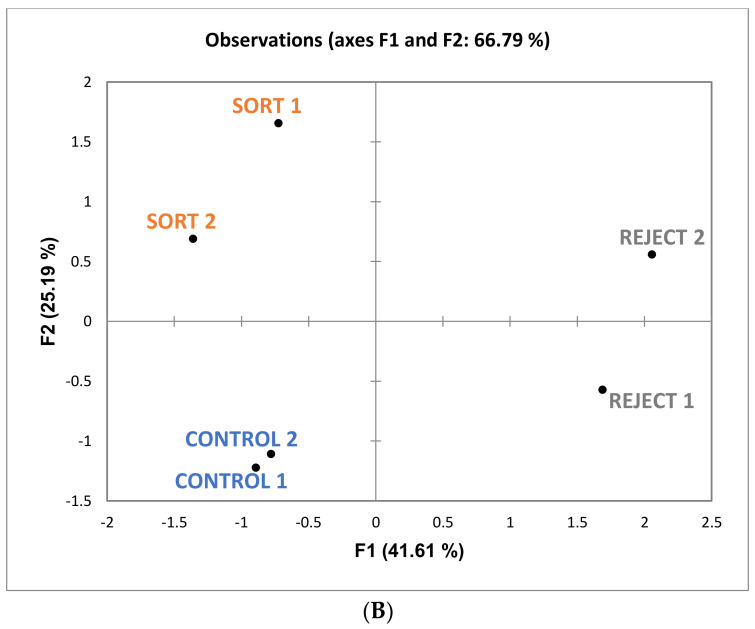
Loadings (**A**) and score (**B**) plots of a multiple factor analysis (MFA) between volatile compounds (blue) in Barbera wines that differed significantly, and all sensory attributes (red) generated from the respective descriptive analysis panel. Only biological replications consistent with those for sensory were included in the MFA.

**Figure 9:**
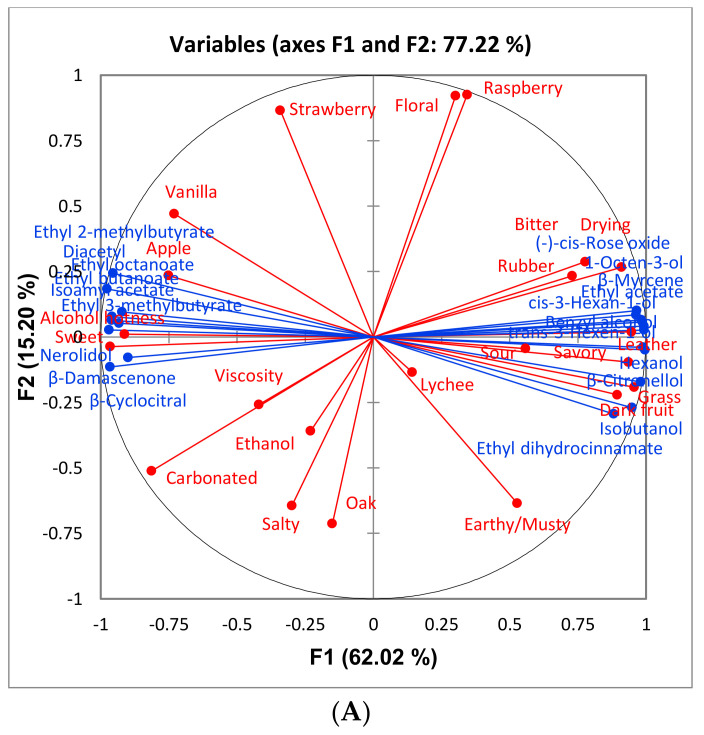

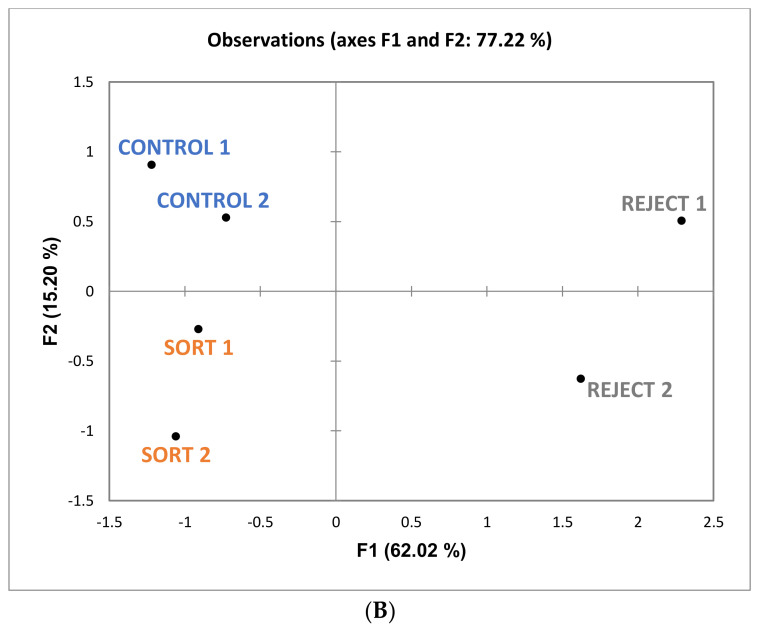
Loadings (**A**) and score (**B**) plots of a multiple factor analysis (MFA) between volatile compounds (blue) in Cabernet Sauvignon wines that differed significantly, and all sensory attributes (red) generated from the respective descriptive analysis panel. Only biological replications consistent with those for sensory were included in the MFA.

Multiple Factor Analysis (MFA) was performed for each variety using all sensory attributes and only volatile compounds that differed significantly among treatments (Figure 7, Figure 8 and Figure 9). This was done to observe the association, if any, of the significant volatile compounds and sensory attributes. For GN wines, the only significant attribute was “SO2”. From Figure 7, isobutanol, which can impart a solventlike aroma in wine, is grouped closely with “SO2”. It is possible that wines with a higher isobutanol concentration were perceived to be higher in “SO2” aroma. For BA wines, there does not appear to be a trend among sensory attributes and volatile compounds (Figure 8). For CS wines, “apple” is grouped closely with ethyl esters (ethyl 3-methylbutyrate, ethyl 2-methylbutyrate, ethyl butanoate, and ethyl octanoate), which provides evidence that this may have caused the increased perception of this attribute in the control and sort treatments (Figure 9).

Overall, optical sorting had minimal impact on the sensory properties of the three varieties tested. It is possible that the chemical differences noted earlier were too small to result in consistent differences by descriptive analysis. Even though the wines made from reject material contained significantly higher concentrations of higher alcohols, it did not result in a difference in sensory perception. Higher alcohols have a relatively high sensory threshold (ranging from around 0.4–40 mg/L depending on the specific compound and the wine medium [20]). It is possible that the concentration of these compounds in the reject wines was below the sensory threshold.

## 4. Discussion

The purpose of this study was to determine what effects, if any, optical berry sorting had on wine made from different red grape varieties, and to investigate the potential to use optical sorters to sort for different ripeness levels using color as a main criterion. Given the observed differences in Brix and final ethanol content, optical sorting seemed to be successful in removing underripe berries for CS and possibly for BA; however, this did not result in a significant difference in the final ethanol content between the sort and control treatments. The removal of underripe berries was also evident by the difference in color among treatments. For BA, the rejected treatments were significantly lighter in color; however, the color of the sort and control treatments was very similar, whereas a similar trend was observed in the CS treatments. Wines made from GN generally did not follow these trends; possibly because sorting parameters were too aggressive for this cultivar, resulting in a high percent rejection of optimal berries. This may have minimized potential differences between reject wine with the other treatments. Another possibility is that color differences in the GN fruit did not correspond to differences in sugar content. From these results, it may be concluded that, when using color as a criterion, optical sorting based on ripeness level was successful but may be dependent on variety and fruit variability. Additionally, the impact on the resulting wine is likely dependent on the initial variability in grape ripeness. The optical sorter was successful in removing MOG. This result was reflected in the phenolic analyses; reject treatments were generally higher in total phenolics and tannin, most likely due to the greater proportion of MOG included in the must. The decrease in anthocyanins is likely due to the higher percentage of green, underripe berries in the reject treatment musts. A study that made wine with the addition of MOG found that this addition significantly increased the phenolic and tannin content in the resulting wines [21]. Despite the differences observed in the phenolic composition of the reject wines, the control and sort treatments were very similar for all three varieties. This is in contrast with some previous studies that have found wine made from optical sorted fruit had significantly different levels of phenolics. One study found that optical sorting led to wines with higher levels of total phenolics [5]. It should be mentioned that the researchers here did whole cluster pressing for their control wines (Chardonnay), whereas the sorted wines were destemmed. It is possible that higher levels of phenolics were extracted due to the damage caused by the destemming process on the seeds and skins. Another study found that wine made from optically sorted grapes that were machine harvested generally had lower levels of phenolics; levels that were similar to the same wines made from a handpick treatment [6]. Given that the rejects were, in general, significantly higher in total phenolics and tannin than the control and sort treatments, it can be suggested that optical sorting has the potential to decrease the phenolic content in wine; however, there was not enough MOG to show a large impact in the current study. Optical sorting likely has a greater impact on mechanically harvest fruit due to generally higher levels of MOG observed from this harvest method. 

Some differences were found among treatments in the aroma profiles of the wines. Few compounds differed significantly between sort and control treatment and, in general, the reject treatments had greater concentrations of higher alcohols and control and sort treatments had greater concentrations of ethyl esters. The higher ethanol content of the sort and control treatments as well as their lower pH (Table 5) can lead to a higher production of esters [15]. In general, reject treatments contained significantly more suspended solids then the control and sort treatments for all varieties studied. Research has shown that high levels of suspended solids during fermentation can lead to greater production of higher alcohols [16,17,18]. 

Descriptive analysis indicated only one significantly different attribute among GN treatments and only two significantly different attributes among BA treatments. BA control and sort wines were associated with the “alcohol” descriptor which correlated with the higher ethanol levels in these treatments compared to the reject treatment. Similarly, there were only three significant attributes among the CS treatments. “Alcohol hotness” related to ethanol content as previously described. The control and sort treatments were also rated significantly higher in “apple” and “sweet” aromas compared to the reject treatment. Some studies have shown that higher levels of ethanol can increase the perception of sweetness in a wine [22,23]. However, as King et al. [24] noted, there is disagreement in this regard, as other studies have shown that ethanol content can either decrease or have no effect on the perception of sweetness [25,26]. Thus, this may not be a sufficient explanation as to why the control and sort wines were rated significantly higher in sweetness. Perhaps the higher concentration of total phenolics and tannin in reject wines could explain the difference (Table 6) given that phenolics in wine contribute to bitterness and astringency. From the PCA in Figure 6, it can be noted that “bitter” and “drying” (with “drying” being a component of astringency) are more associated with reject wines. Although these attributes are not significantly different among the treatments there appears to be a trend which could impact the perception of sweetness. One study found that increasing bitterness in coffee (by the addition of caffeine) decreased the perception of sweetness [27]. It is possible that reject wines were rated lower in “sweet” due to the higher concentration of phenolic compounds (which can contribute to bitterness perception) thus decreasing the perception of sweetness. The higher perception of sweetness in the control and sort wines may also be attributed to the higher intensity of the “apple” aroma, which the judges could have associated with a sweet taste. One study found that retronasal aroma perception of fruity compounds increased with an increasing level of sweetness in a model wine solution [28]. The authors also noted several other studies which found that aroma compounds can enhance the perception of sweetness in different foods and beverages. Another study found that samples described as “fruity” were also often associated with a “sweet” aroma [29]. This provides further evidence that the judges in the current study may have associated these attributes together. The overall sensory differences were minimal, and the wines were determined to be similar. 

The results from this study largely agree with results from previous studies investigating the effects of optical sorters. It is possible that there was not enough variation in the starting material of the current study for optical sorting to have a large impact. Optical sorters may be used to greater effect during vintages with inconsistent ripening, issues with raisining, or large amounts of berry damage, possibly caused by either birds and/or fungal infections. Future research should investigate the impact of optical sorters in these scenarios. 

## Figures and Tables

**Table 1:** Attributes and corresponding reference standards for the Grenache descriptive analysis panel.

Attribute	Reference Standard
Aroma	
Citrus	2 × 1 cm slice each of grapefruit and orange skin
Pear	20 mL pear juice (Santa Cruz)
Red Fruit	0.5 g frozen sliced strawberries (Market Pantry) + 1 Tbsp ^a^ raspberry preserves (Trader Joe’s)
Apple	15 g thawed frozen green apple (Save Mart) + 2 Tbsp apple sauce + 40 mL base wine ^b^
Raisin	3 Thompson seedless raisins (Trader Joe’s)
Dark Fruit	3 thawed and crushed frozen blackberries (Best Yet)
Caramel	One Tbsp caramel sauce (Trader Joe’s)
Herbal	One pinch thyme (McCormick) + one pinch sage (McCormick)
Earthy	1 Tbsp soil from vineyard + 1 Tbsp potting soil
Oak	1 g enological tannin + 20 mL base wine
SO2	0.25 mL 15% potassium metabisulfite solution + 10 mL water
Ethanol	20 mL Everclear (75.5% *v*/*v*) + 30 mL base wine
Taste	
Sour	3 g/L L-(+)-tartaric acid (Sigma-Aldrich) in water
Bitter	0.8 g/L caffeine (Sigma-Aldrich) in water
Sweet	15 g/L pure cane sugar (C&H) in water
Salty	3 g/L salt (Morton Kosher Salt) in water
Mouthfeel	
Viscosity	5 g/L carboxymethylcellulose (Sigma-Aldrich) in water
Burning	250 mL/L vodka (Seagram’s Extra Smooth, 40% *v*/*v*) in water
Sparkling	Sparkling water (Crystal Geyser)
Astringent	0.8 g/L alum (McCormick) in water

^a^ Tablespoon abbreviated as Tbsp. ^b^ Franzia Cabernet Sauvignon was used as the base wine.

**Table 2:** Attributes and corresponding reference standards for the Barbera descriptive analysis panel.

Attribute	Reference Standard
Aroma	
Dark fruit	3 thawed and crushed frozen blackberries (Best Yet)
Cherry	3 thawed and crushed frozen black cherries (Dole) + 10 mL black cherry juice (R.W. Knudsen)
Cranberry	6 thawed and crushed frozen cranberries in 20 mL Cranberry juice (Simply Cranberry Cocktail)
Raspberry	1 Tbsp ^a^ raspberry preserves (Trader Joe’s)
Fresh Banana	2 × 1 cm circle of fresh banana, no peel
Fresh veg	10 g Frozen Sliced Green Beans (McCains) + 10 g Frozen Green Peas (McCains) + 1 g bell pepper
Tomato	6 g cut fresh tomato (Safeway)
Black pepper	1/8 teaspoon cracked black pepper
Leather	5 (1’’) brown leather shoelace strips (Kiwi Outdoor) + 30 mL base wine ^b^
Vanilla	2 mL vanilla extract (McCormick) + 25 mL wine
Musty/Earthy	0.5 g dried portobello mushroom + 1 Tbsp potting soil
Smoke	4 drops liquid smoke (Colgin) + 40 mL base wine
Oak	1 American Oak cube (M+) + 1 French Oak Cube (Light) + 1 French Oak Cube (M) (Innerstave Cube Trail Kit) + 20 mL base wine
Alcohol	20 mL Everclear (75.5% *v*/*v*) + 30 mL base wine
Taste	
Sour	3.5 g/L L-(+)-tartaric acid (Sigma-Aldrich) in water
Bitter	1.5 g/L caffeine (Sigma-Aldrich) in water
Sweet	15 g/L pure cane sugar (C&H) in water
Salty	2 g/L salt (Morton Kosher Salt) in water
Mouthfeel	
Smooth	Water
Effervescent	Sparkling water (Crystal Geyser)
Viscous	3 g/L carboxymethylcellulose (Sigma-Aldrich) in water
Puckery	200 mL/L white vinegar (365 Everyday Value) in water
Alcohol hotness	250 mL/L vodka (Seagram’s Extra Smooth, 40% *v*/*v*) in water
Dry	1.3 g/L alum (McCormick) in water
Grippy	Drawing or tightening sensation felt in the mouth, lips and/or cheeks, lack of slip between mouth surfaces resulting in the inability to easily move mouth surfaces across each other (Definition)
Prickling	A coarse irritation typified by exposure to acetic acid (Definition)

^a^ Tablespoon abbreviated as Tbsp. ^b^ Franzia Cabernet Sauvignon was used as the base wine.

**Table 3:** Attributes and corresponding reference standards for the Cabernet Sauvignon descriptive analysis panel.

Attribute	Reference Standard
Aroma	
Apple	20 mL Apple juice (R.W. Knudsen)
Dark fruit	3 thawed and crushed frozen blackberries (Save Mart) + 3 thawed and crushed frozen black cherries (365 Everyday Value)
Strawberry	1 g frozen sliced strawberries (Market Pantry)
Raspberry	1 Tbsp ^a^ raspberry preserves (Trader Joe’s)
Lychee	1 whole peeled lychee (Dynasty)
Vanilla	2 drops vanilla extract (McCormick)
Floral	1 drop of violet syrup (Monin) + 1 drop of lavender oil (Simplers Botanicals) in 100 mL water, use 5 mL of this solution
Oak	1 American Oak cube (M+) + 1 French Oak Cube (Light) (Innerstave Cube Trail Kit) + 20 mL base wine ^b^
Leather	5 (1’’) brown leather shoelace strips (Kiwi Outdoor) + 20 mL base wine
Earthy/Musty	2 g dried portabello mushroom + 1 Tbsp potting soil + a few drops of water
Grass	30 (1”) blades of fresh grass, rolled/crushed between fingers + 30 mL base wine
Rubber	4 (1’’) cut rubber bands pieces + 20 mL base wine
Ethanol	20 mL Everclear (75.5% *v*/*v*) + 30 mL base wine
Taste	
Sweet	20 g/L pure cane sugar (C&H) in water
Salty	3.5 g/L salt (Morton Kosher Salt) in water
Savory	5 g/L MSG (Accent Seasoning) in water
Sour	2.2 g/L L-(+)-tartaric acid (Sigma-Aldrich) in water
Bitter	0.8 g/L caffeine (Sigma-Aldrich) in water
Mouthfeel	
Viscosity	4 g/L carboxymethylcellulose (Sigma-Aldrich) in water
Carbonated	Sparkling water (Crystal Geyser)
Alcohol hotness	300 mL/L Vodka (Seagram’s Extra Smooth, 40% *v*/*v*) in water
Drying	1.3 g/L alum (McCormick) in water

^a^ Tablespoon abbreviated as Tbsp. ^b^ Franzia Cabernet Sauvignon was used as the base wine.

**Table 4:** Means and standard deviations for Brix, pH, titratable acidity (TA), yeast assimilable nitrogen (YAN), and malic acid of musts taken after processing and mixing and before any additions were made.

	Treatment	Brix	pH	TA (g/L Tartaric Acid)	YAN (mg/L)	Malic Acid (mg/L)
GN	Control	22.9 ± 1.2 a	3.55 ± 0.04 a	3.5 ± 0.2 a	131 ± 4.1 a	529 ± 31.9 a
	Sort	23.3 ± 1.5 a	3.54 ± 0.03 a	3.6 ± 0.1 a	142 ± 18.2 a	611 ± 34.4 a
	Reject	23.3 ± 0.0 a	3.51 ± 0.00 a	4.1 ± 0.0 b	156 ± 5.2 a	815 ± 79.2 b
BA	Control	25.4 ± 0.4 a	3.22 ± 0.00 a	6.9 ± 0.1 a	312 ± 73.7 a	1856 ± 50.5 a
	Sort	25.9 ± 0.2 a	3.27 ± 0.06 a	6.7 ± 0.4 a	303 ± 18.6 a	2067 ± 178.4 a
	Reject	24.8 ± 1.6 a	3.25 ± 0.00 a	7.4 ± 0.1 a	290 ± 28.7 a	2638 ± 33.2 b
CS	Control	23.3 ± 0.1 a	3.67 ± 0.01 a	3.5 ± 0.1 a	134 ± 4.0 a	932 ± 27.4 a
	Sort	23.7 ± 0.3 b	3.71 ± 0.01 b	3.7 ± 0.1 a	139 ± 5.7 a	1156 ± 275.1 a
	Reject	22.7 ± 0.1 c	3.79 ± 0.02 c	4.1 ± 0.2 b	135 ± 11.0 a	1970 ± 262.3 b

Different letters in the same column per variety indicate significance at *p* < 0.05 (*n* = 3 for sort and control treatments, *n* = 2 for reject treatment).

**Table 5:** Means and standard deviations for basic wine chemistry analysis performed at the time of bottling.

	Treatment	% EtOH (*v*/*v*)	pH	TA (g/L Tartaric Acid)	RS (g/L)	VA (g/L)
GN	Control	13.8 ± 0.2 a	3.31 ± 0.05 a	6.5 ± 0.1 a	0.19 ± 0.02 a	0.2 ± 0.0 a
	Sort	13.3 ± 0.4 a	3.32 ± 0.04 a	5.6 ± 0.2 b	0.18 ± 0.02 a	0.3 ± 0.1 a
	Reject	12.9 ± 0.4 a	3.39 ± 0.02 a	5.9 ± 0.3 b	0.16 ± 0.01 a	0.3 ± 0.0 a
BA	Control	14.9 ± 0.2 a	3.18 ± 0.02 a	8.8 ± 0.1 a	0.43 ± 0.01 a	0.3 ± 0.0 a
	Sort	15.2 ± 0.4 a	3.24 ± 0.05 a	8.1 ± 0.7 a	0.45 ± 0.02 a	0.3 ± 0.0 a
	Reject	13.5 ± 0.1 b	3.32 ± 0.03 b	8.1 ± 0.1 a	0.38 ± 0.01 b	0.4 ± 0.0 b
CS	Control	13.2 ± 0.0 a	3.19 ± 0.02 a	6.8 ± 0.1 a	0.13 ± 0.01 a	0.2 ± 0.0 a
	Sort	13.4 ± 0.2 a	3.31 ± 0.02 b	6.3 ± 0.1 b	0.12 ± 0.01 a	0.2 ± 0.1 a
	Reject	11.8 ± 0.7 b	3.57 ± 0.01 c	7.0 ± 0.4 a	0.16 ± 0.04 a	0.6 ± 0.2 b

Different letters in the same column per variety indicate significance at *p* < 0.05 (*n* = 3 for sort and control treatments, *n* = 2 for reject treatment). VA (volatile acidity) are expressed as g/L acetic acid.

**Table 6:** Means and standard deviations for total phenolics, tannin, and anthocyanin (mg/L) in finished wines determined by the Adams-Harbertson assay.

	Treatment	Total Phenolics (mg/L)	Tannin (mg/L)	Anthocyanin (mg/L)
GN	Control	834.3 ± 10.8 a	80.2 ± 9.4 a	128.5 ± 16.8 a
	Sort	753.3 ± 17.0 b	85.1 ± 13.9 a	112.9 ± 5.0 a
	Reject	804.6 ± 29.9 a	99.4 ± 12.2 a	71.1 ± 0.2 b
BA	Control	811.6 ± 97.6 a	11.8 ± 1.2 a	232.1 ± 7.9 a
	Sort	871.6 ± 39.3 a	10.5 ± 5.2 a	257.6 ± 13 a
	Reject	1117.5 ± 105.6 b	51.9 ± 2.7 b	98 ± 18.9 b
CS	Control	1488.2 ± 21.9 a	184.7 ± 7.6 a	518.9 ± 11.7 a
	Sort	1468.7 ± 61.4 a	175.8 ± 12.3 a	518.5 ± 24.5 a
	Reject	2464.3 ± 238.7 b	382.2 ± 24.3 b	131.3 ± 59.8 b

Different letters in the same column per variety indicate significance at *p* < 0.05 (*n* = 3 for sort and control treatments, *n* = 2 for reject treatment).

**Table 7:** Concentration (mg/L) of phenolic compounds in Grenache wines determined by reverse-phase high-performance liquid chromatography (RP-HPLC) from samples taken around six months after bottling.

Compound	GN		
	Control	Sort	Reject
Gallic acid	9.50 ± 0.58 a	8.32 ± 0.32 b	14.25 ± 0.12 c
(+)-Catechin	21.10 ± 0.39 a	19.51 ± 0.72 b	24.07 ± 0.75 c
B1	16.62 ± 1.60 a	17.75 ± 1.11 a	18.92 ± 0.89 a
(−)-Epicatechin	0.38 ± 0.05 a	0.32 ± 0.03 a	0.37 ± 0.06 a
Epicatgallate	1.62 ± 0.37 a	1.27 ± 0.28 a	1.67 ± 0.12 a
Polymeric phenols	66.70 ± 4.19 a	50.50 ± 5.93 a	59.07 ± 0.27 a
Caftaric acid	42.19 ± 5.69 a	24.47 ± 6.16 b	13.24 ± 1.76 b
Caffeic acid	21.54 ± 4.88 a	29.21 ± 4.68 a	44.04 ± 1.76 b
Coutaric acid	10.18 ± 1.37 a	6.01 ± 1.22 b	3.54 ± 0.38 b
p-Coumaric acid	4.43 ± 1.16 a	5.94 ± 1.22 a	8.50 ± 0.09 b
Quer-galactoside	1.19 ± 0.09 a	0.87 ± 0.02 b	0.84 ± 0.06 b
Quer-3-glucoside	5.95 ± 1.25 a	4.59 ± 0.09 a	2.53 ± 0.30 b
Quer-glucuronide	11.25 ± 2.84 a	7.55 ± 0.98 a	10.72 ± 1.82 a
Quer-rhamnoside	4.23 ± 0.42 a	3.21 ± 0.10 b	2.48 ± 0.42 b
Quercetin	4.02 ± 0.79 a	3.69 ± 0.11 a	3.44 ± 0.24 a
Delph-3-gluc	1.24 ± 0.15 a	1.03 ± 0.04 b	0.58 ± 0.07 c
Pet-3-gluc	2.53 ± 0.23 a	2.11 ± 0.06 b	1.17 ± 0.09 c
Peo-3-gluc	2.81 ± 0.14 a	2.27 ± 0.08 b	1.49 ± 0.09 c
Malv-3-gluc	71.92 ± 6.41 a	64.86 ± 4.64 a	38.58 ± 0.63 b
Pet-3-acetylgluc	-	-	-
Malv-3-acetylgluc	2.72 ± 0.93 a	2.27 ± 0.10 a	1.90 ± 0.24 a
Malv-3-p-coumgluc	7.02 ± 0.77 a	6.71 ± 0.73 a	3.22 ± 0.02 b
Polymeric pigments	3.96 ± 0.30 a	2.73 ± 0.23 b	3.05 ± 0.05 b
Flavan-3-ols	46.54 ± 1.94 a	41.75 ± 2.29 b	51.38 ± 2.08 a
Hydroxycinnamic acids	78.33 ± 3.64 a	65.64 ± 1.78 b	69.31 ± 3.46 b
Flavonols	26.64 ± 1.14 a	19.92 ± 1.19 b	20.01 ± 2.35 b
Anthocyanins	88.25 ± 8.05 a	79.25 ± 5.25 a	46.94 ± 1.14 b

Treatments with different letters indicate significance at *p* < 0.05 (*n* = 3 for sort and control treatments, *n* = 2 for reject treatment).

**Table 8:** Concentration (mg/L) of phenolic compounds in Barbera wines determined by RP-HPLC from samples taken around six months after bottling.

Compound	BA		
	Control	Sort	Reject
Gallic acid	12.82 ± 0.74 a	15.08 ± 0.55 b	35.15 ± 0.39 c
(+)-Catechin	11.13 ± 0.52 a	11.42 ± 0.72 a	32.72 ± 2.80 b
B1	28.45 ± 0.95 a	27.01 ± 2.43 a	51.14 ± 4.31 b
(−)-Epicatechin	0.43 ± 0.01 a	0.48 ± 0.04 a	0.95 ± 0.11 b
Epicatgallate	2.89 ± 0.29 a	2.91 ± 0.70 a	2.31 ± 0.06 a
Polymeric phenols	134.60 ± 25.35 a	131.70 ± 8.99 a	136.72 ± 5.24 a
Caftaric acid	52.86 ± 8.82 a	56.55 ± 3.76 a	12.69 ± 1.95 b
Caffeic acid	9.74 ± 2.14 a	12.77 ± 0.57 b	55.76 ± 0.39 c
Coutaric acid	21.52 ± 3.18 a	23.09 ± 1.54 a	5.57 ± 0.53 b
p-Coumaric acid	3.25 ± 0.64 a	4.25 ± 0.10 b	16.23 ± 0.10 c
Quer-galactoside	2.37 ± 0.16 a	2.93 ± 0.36 b	2.07 ± 0.06 a
Quer-3-glucoside	4.68 ± 0.71 a	8.72 ± 2.19 b	2.32 ± 0.54 a
Quer-glucuronide	24.38 ± 2.07 a	27.44 ± 0.86 a	24.97 ± 1.70 a
Quer-rhamnoside	2.68 ± 0.26 a	3.15 ± 0.12 b	1.41 ± 0.11 c
Quercetin	7.78 ± 0.60 a	7.58 ± 0.38 a	8.40 ± 0.46 a
Delph-3-gluc	6.49 ± 0.36 a	7.47 ± 0.83 a	2.47 ± 0.65 b
Pet-3-gluc	11.52 ± 0.28 a	13.02 ± 1.23 a	4.19 ± 1.10 b
Peo-3-gluc	3.66 ± 0.23 a	4.35 ± 0.22 b	1.48 ± 0.35 c
Malv-3-gluc	76.77 ± 1.23 a	83.73 ± 4.22 a	27.02 ± 7.84 b
Pet-3-acetylgluc	2.24 ± 0.05 a	2.74 ± 0.48 a	0.93 ± 0.22 b
Malv-3-acetylgluc	15.14 ± 0.62 a	17.04 ± 2.30 a	7.00 ± 1.39 b
Malv-3-p-coumgluc	10.27 ± 0.15 a	10.64 ± 0.82 a	3.32 ± 1.27 b
Polymeric pigments	12.14 ± 2.88 a	10.99 ± 1.62 a	8.24 ± 0.88 a
Flavan-3-ols	44.96 ± 1.72 a	43.74 ± 2.98 a	87.05 ± 7.08 b
Hydroxycinnamic acids	87.37 ± 9.50 a	91.82 ± 3.43 a	90.26 ± 2.97 a
Flavonols	41.89 ± 3.79 a	49.82 ± 3.88 b	39.18 ± 2.88 a
Anthocyanins	126.09 ± 1.00 a	139.00 ± 9.88 a	46.40 ± 12.82 b

Treatments with different letters indicate significance at *p* < 0.05 (*n* = 3 for sort and control treatments, *n* = 2 for reject treatment).

**Table 9:** Concentration (mg/L) of phenolic compounds in Cabernet Sauvignon wines determined by RP-HPLC from samples taken around six months after bottling.

Compound	CS		
	Control	Sort	Reject
Gallic acid	7.86 ± 0.19 a	9.00 ± 0.76 a	42.34 ± 10.61 b
(+)-Catechin	17.96 ± 0.79 a	20.79 ± 1.86 a	68.90 ± 3.91 b
B1	32.21 ± 0.93 a	32.37 ± 0.98 a	73.50 ± 5.60 b
(−)-Epicatechin	1.40 ± 0.02 a	1.49 ± 0.02 a	0.95 ± 0.12 b
Epicatgallate	7.31 ± 0.23 a	6.95 ± 0.30 a	5.27 ± 0.78 b
Polymeric phenols	260.54 ± 6.35 a	249.64 ± 18.30 a	333.24 ± 70.73 a
Caftaric acid	3.58 ± 0.95 a	2.55 ± 0.67 a	-
Caffeic acid	26.84 ± 0.74 a	27.89 ± 1.14 a	20.09 ± 1.01 b
Coutaric acid	1.62 ± 0.29 a	2.20 ± 0.30 a	-
p-Coumaric acid	9.88 ± 0.19 a	9.71 ± 0.39 a	5.03 ± 0.08 b
Quer-galactoside	4.24 ± 0.03 a	4.25 ± 0.05 a	1.91 ± 0.25 b
Quer-3-glucoside	12.48 ± 0.19 a	10.55 ± 0.61 b	2.58 ± 0.34 c
Quer-glucuronide	22.61 ± 0.21 a	20.24 ± 0.37 a	25.81 ± 2.60 a
Quer-rhamnoside	14.94 ± 0.17 a	13.26 ± 0.24 b	4.42 ± 1.04 c
Quercetin	3.94 ± 0.04 a	4.36 ± 0.17 b	4.84 ± 0.13 c
Delph-3-gluc	7.05 ± 0.13 a	6.92 ± 0.35 a	1.12 ± 0.74 b
Pet-3-gluc	9.43 ± 0.27 a	9.53 ± 0.49 a	1.61 ± 1.14 b
Peo-3-gluc	5.44 ± 0.15 a	5.04 ± 0.10 a	1.05 ± 0.67 b
Malv-3-gluc	185.27 ± 2.76 a	186.77 ± 9.01 a	32.51 ± 22.17 b
Pet-3-acetylgluc	3.03 ± 0.04 a	3.27 ± 0.16 a	0.68 ± 0.42 b
Malv-3-acetylgluc	77.66 ± 1.93 a	79.39 ± 4.57 a	17.64 ± 10.61 b
Malv-3-p-coumgluc	25.64 ± 0.44 a	24.02 ± 1.37 a	2.58 ± 1.89 b
Polymeric pigments	19.37 ± 0.39 a	19.00 ± 1.00 a	13.67 ± 3.34 b
Flavan-3-ols	58.88 ± 1.16 a	61.60 ± 3.30 a	148.62 ± 10.42 b
Hydroxycinnamic acids	42.51 ± 2.09 a	41.76 ± 0.75 a	25.13 ± 1.09 b
Flavonols	58.20 ± 0.44 a	52.66 ± 1.04 b	39.55 ± 1.10 c
Anthocyanins	313.51 ± 4.90 a	314.94 ± 15.97 a	57.19 ± 38.23 b

Treatments with different letters indicate significance at *p* < 0.05 (*n* = 3 for sort and control treatments, *n* = 2 for reject treatment).

**Table 10:** Mean color scores from sensory analysis. Judges were instructed to match wine colors to colors on a poster (Les Couleurs Du Vin, Bouchard Aîné & Fils). Higher scores corresponded to darker colors.

	Score		
Variety	Control	Sort	Reject
GN	20.4 a	16.5 b	15.6 b
BA	33.6 a	32.7 a	26.3 b
CS	33.6 a	33.4 a	30.9 a

Treatments with different letters indicate significance at *p* < 0.05 (*n* = 11 for GN, *n* = 10 for BA and CS).

**Table 11:** Wine colors determined from CIELAB values measured using a CR-400 Chroma Meter within 6 months of sensory analysis. CIELAB values were converted to colors using Colorizer.org.

	Color		
Variety	Control	Sort	Reject
GN			
BA			
CS			

## Data Availability

The data presented in this study are available on request from the corresponding author. The data are not publicly available due to delay in open access availability of thesis.

## Supplementary Materials

The following are available online at https://www.mdpi.com/2304-8158/10/2/402/s1, Figure S1: Biplot of BA treatments including all ethyl ester and higher alcohols. A correlation matrix was used; Figure S2: Cobweb plot of mean intensity scores from descriptive analysis of Grenache wines. An asterisk indicates compounds that differed significantly among treatments (*n* = 11, *p* < 0.05); Figure S3: Cobweb plot of mean intensity scores from descriptive analysis of Barbera wines. Asterisk indicates attributes that differed significantly among treatments (*n* = 10, *p* < 0.05); Figure S4: Cobweb plot of mean intensity scores from descriptive analysis of Barbera wines. Asterisk indicates attributes that differed significantly among treatments (*n* = 10, *p* < 0.05); Table S1: Compounds measured by HS-SPME-GC-MS analysis of wines with CAS number, retention time, and ions chosen for selected ion monitoring (SIM); Table S2: MANOVA results from descriptive analysis of GN wines; Table S3: MANOVA results from descriptive analysis of CS wines; Table S4: MANOVA results from descriptive analysis of CS wines.
