# Supplementary material for: The Impact of Optical Berry Sorting on Red Wine Composition and Sensory Properties
Source: Foods. 2021 Feb 12;10(2):402. doi: 10.3390/foods10020402 (PMC7917668; doi:10.3390/foods10020402)
Supplement: Supplementary file 1 [file foods-10-00402-s001.pdf]

Supplementary Materials: The following are available online at www.mdpi.com/xxx/s1, Figure S1: Biplot of BA treatments including all ethyl ester and higher alcohols. A correlation matrix was used; S2: Cobweb plot of mean intensity scores from descriptive analysis of Grenache wines. An asterisk indicates compounds that differed significantly among treatments (n = 11, p < 0.05); S3: Cobweb plot of mean intensity scores from descriptive analysis of Barbera wines. Asterisk indicates attributes that differed significantly among treatments (n = 10, p < 0.05); S4: Cobweb plot of mean intensity scores from descriptive analysis of Barbera wines. Asterisk indicates attributes that differed significantly among treatments (n = 10, p < 0.05); Table S1: Compounds measured by HS-SPME-GC-MS analysis of wines with CAS number, retention time, and ions chosen for selected ion monitoring (SIM); S2: MANOVA results from descriptive analysis of GN wines; S3: MANOVA results from descriptive analysis of CS wines; S4: MANOVA results from descriptive analysis of CS wines.

Table S1. Compounds measured by HS-SPME-GC-MS analysis of wines with CAS number, retention time, and ions chosen for selected ion monitoring (SIM).

Compound	CAS #	Retention Time (min)	SIM Ions
Ethyl acetate	141-78-6	3.053	43, 61, 88
Ethyl isobutyrate	97-62-1	4.443	43, 71, 86, 116
Diacetyl	431-03-8	4.782	43, 71, 86, 116
Ethyl butanoate	105-54-4	6.508	71, 88, 116
Ethyl 2-methylbutyrate	7452-79-1	7.046	57, 102, 130
Ethyl 3-methylbutyrate	108-64-5	7.632	55, 88, 130
Isobutanol	78-83-1	8.717	43, 55, 74
Isoamyl acetate	123-92-2	9.895	55, 87, 130
β-Myrcene	123-35-3	11.639	69, 93, 136
α-Terpinene	99-86-5	12.080	93, 121, 136
Limonene	138-86-3	12.956	68, 93, 136
Isoamyl alcohol	123-51-3	13.856	57, 70, 88
Ethyl hexanoate	123-66-0	15.076	88, 115, 144
ϱ-Cymene	99-87-6	16.485	91, 119, 134
Hexyl acetate	142-13-0	16.931	43, 45, 56, 84, 88, 101
Acetoin	513-86-0	17.231	43, 45, 56, 84, 88, 101
Ethyl lactate	687-47-8	20.201	45, 75
(-)-cis-Rose oxide	16409-43-1	20.460	69, 139, 154
Hexanol	111-27-3	20.701	69, 84, 101
trans-3-Hexen-1-ol	928-97-2	21.167	41, 57, 67, 82, 100
cis-3-Hexen-1-ol	928-96-1	22.087	41, 57, 67, 82, 100
Ethyl octanoate	106-32-1	24.578	88, 101, 172
1-Octen-3-ol*	3391-86-4	25.230	57, 72, 127
Nerol oxide*	1786-08-9	25.902	68, 83, 152
Benzaldehyde	100-52-7	28.164	77, 105, 106
β-Linalool	78-70-6	29.523	71, 93, 154
2-Undecanone (IS)	112-12-9	31.551	58, 71, 170
β-Cyclocitral*	432-25-78	32.194	123, 137, 152

Ethyl decanoate	110-38-3	33.286	88, 105, 200
β-Citronellol	106-22-9	38.264	69, 82, 156
Nerol*	106-25-2	39.458	69, 93, 154
β-Damascenone	23726-93-4	40.135	69, 121, 190
Geraniol	106-24-1	41.238	69, 93, 154
Benzyl alcohol	100-51-6	42.177	79, 91, 104, 107, 108, 178
Ethyl dihydrocinnamate*	2021-28-5	42.556	79, 91, 104, 107, 108, 178
2-Phenethyl alcohol	60-12-8	43.422	65, 91, 122
Nerolidol	40716-66-3	48.208	65, 93, 222
Octanoic acid	124-07-2	48.690	60, 73, 115

*Indicates compounds that were not detected in Grenache wines.

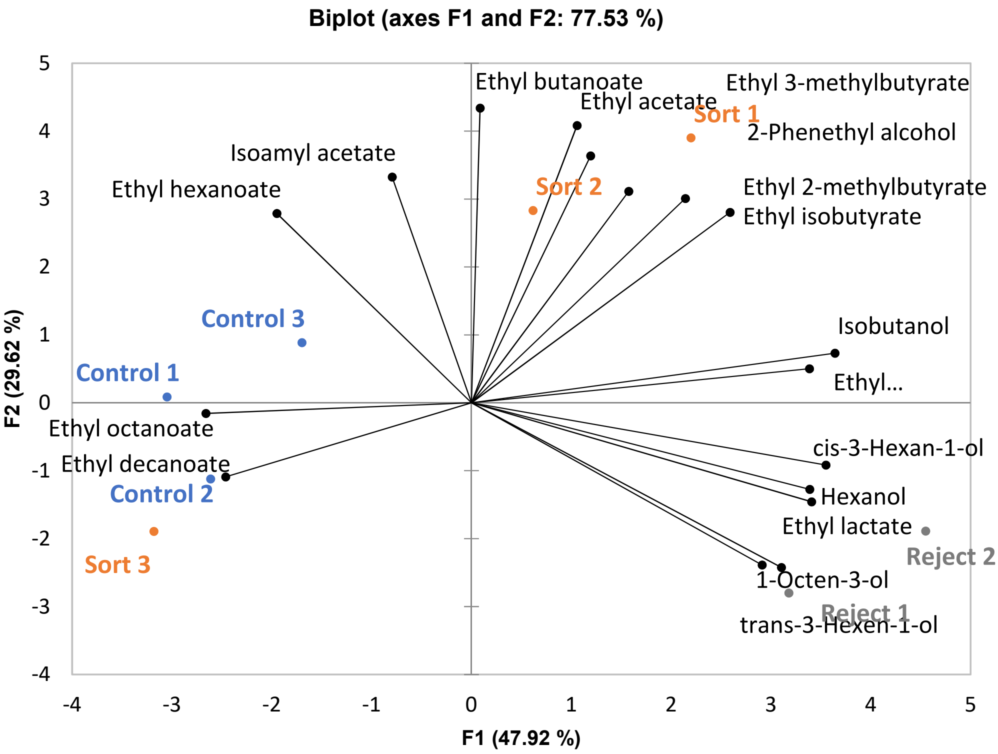

Figure S1. Biplot of BA treatments including all ethyl ester and higher alcohols. A correlation matrix was used.

Table S2. MANOVA results from descriptive analysis of GN wines.

	Judge	Product	Rep
Lambda	0.369	0.914	0.842
F (Observed values)	14.988	0.819	1.640
DF1	20	20	20
DF2	175	175	175
F (Observed values)	1.631	1.631	1.631
p-value	< 0.0001	0.689	0.048

Table S3. MANOVA results from descriptive analysis of BA wines.

	Judge	Product	Rep
Lambda	0.235	0.872	0.774
F (Observed values)	18.863	0.852	1.695
DF1	26	26	26
DF2	151	151	151
F (Observed values)	1.569	1.569	1.569
p-value	< 0.0001	0.673	0.027

Table S4. MANOVA results from descriptive analysis of CS wines.

	Judge	Product	Rep
Lambda	0.679	0.879	0.858
F (Observed values)	3.334	0.967	1.162
DF1	22	22	22
DF2	155	155	155
F (Observed values)	1.611	1.611	1.611
p-value	< 0.0001	0.509	0.290

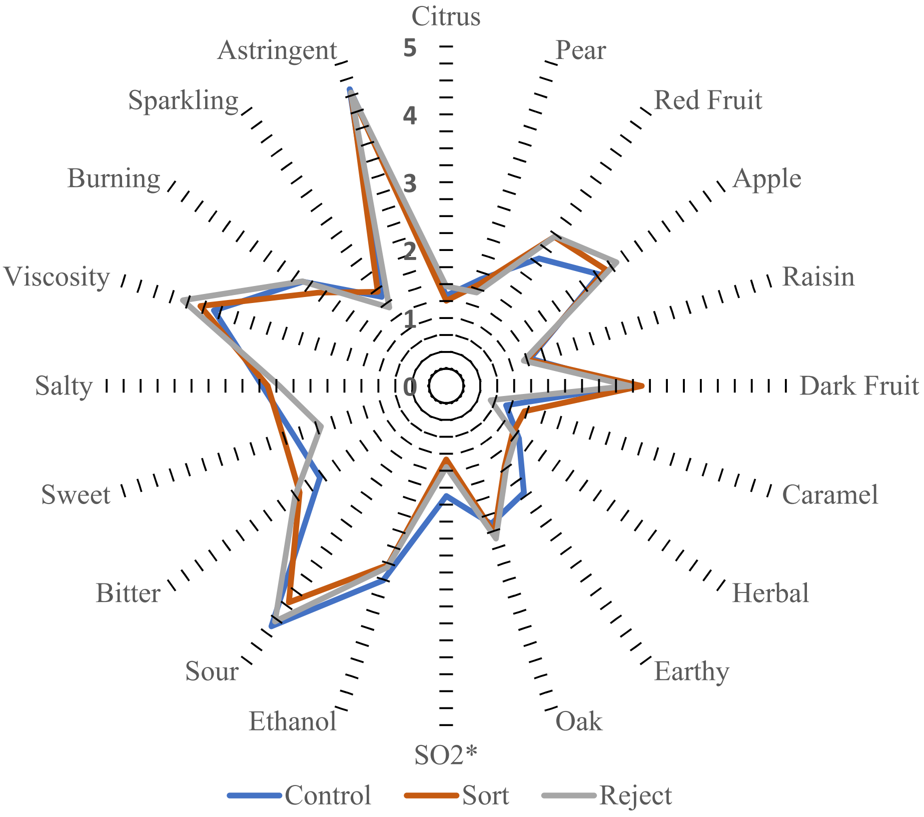

Figure S2. Cobweb plot of mean intensity scores from descriptive analysis of Grenache wines. An asterisk indicates compounds that differed significantly among treatments (n = 11, $p < 0.05$).

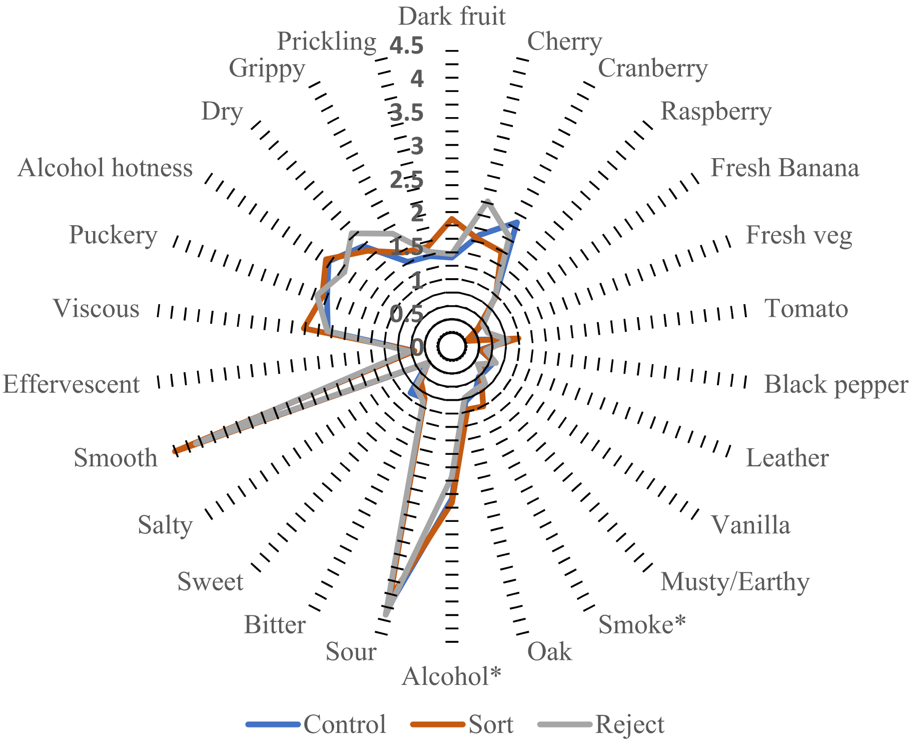

Figure S3. Cobweb plot of mean intensity scores from descriptive analysis of Barbera wines. Asterisk indicates attributes that differed significantly among treatments (n = 10, $p < 0.05$).

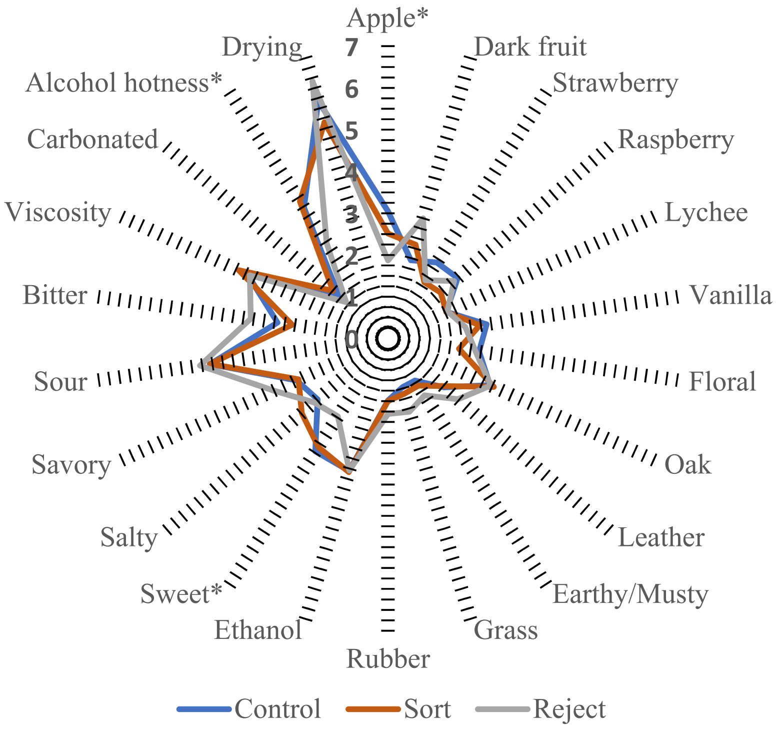

Figure S4. Cobweb plot of mean intensity scores from descriptive analysis of Barbera wines. Asterisk indicates attributes that differed significantly among treatments (n = 10, $p < 0.05$).
